# Formulation development, stability and anticancer efficacy of core-shell cyclodextrin nanocapsules for oral chemotherapy with camptothecin

**DOI:** 10.3762/bjoc.11.22

**Published:** 2015-02-04

**Authors:** Hale Ünal, Naile Öztürk, Erem Bilensoy

**Affiliations:** 1 Division of Nanotechnology and Nanomedicine, Graduate School of Science and Engineering, Hacettepe University, Beytepe, Ankara, 06800, Turkey; 2 Department of Pharmaceutical Technology, Faculty of Pharmacy, Hacettepe University, Sıhhıye, Ankara, 06100, Turkey

**Keywords:** amphiphilic cyclodextrin, camptothecin, core-shell, nanocapsule, oral chemotherapy

## Abstract

**Background:** The aim of this study was to design and evaluate hybrid cyclodextrin (CD) nanocapsules intended for the oral delivery of the anticancer agent camptothecin (CPT) in order to maintain drug stability in the body and to improve its eventual bioavailability. For this reason, an amphiphilic cyclodextrin (CD) derivative per-modified on the primary face 6OCAPRO was used as core molecule to form nanocapsules with the nanoprecipitation technique. Nanocapsules were further coated with the cationic polymer chitosan to improve the cellular uptake and interaction with biological membranes through positive surface charge. Nanocapsules were evaluated for their in vitro characteristics such as particle size, zeta potential, drug loading and release profiles followed by cell culture studies with the MCF-7 and Caco-2 cell line evaluating their anticancer efficacy and permeability. The CD nanocapsules were imaged by scanning electron microscopy (SEM). The concentration of CPT entrapped in nanocapsules was determined by reversed phase HPLC. The in vitro release study of CPT was performed with a dialysis bag method under sink conditions mimicking the gastric and intestinal pH. The hydrolytic stability of CPT in nanocapsules was investigated in simulated gastric and intestinal fluids (SGF, SIF).

**Results:** The mean particle sizes of both anionic and cationic CPT-loaded nanocapsules were in the range of 180–200 nm with polydispersity indices lower than 0.400 indicating monodisperse size distribution of nanocapsules with favourable potential for intracellular drug delivery to tumour cells. Surface charges of anionic and cationic nanocapsules were demonstrated as −21 mV and +18 mV, respectively. The stability of CPT in simulated release media, SGF and SIF were maintained suggesting the improved protection of the drug molecule from rapid hydrolysis degradation or gastrointestinal pH in nanocapsule oily core. Furthermore CD nanocapsules showed higher anticancer efficacy than CPT solution against the MCF-7 cell line. Permeation of CPT across Caco-2 cells was found to be 3 fold higher when incorporated in hybrid CD nanocapsules compared with a DMSO solution.

**Conclusion:** Oral CD nanocapsules indicating increased oral bioavailability might be a promising strategy to maintain the physiological stability and to improve the oral bioavailability of problematic anticancer drugs such as CPT which may contribute to patient quality of life and drug efficacy in cancer therapy.

## Introduction

Cancer is one of the major fatal diseases in the world and causes abnormal growth of cells spreading to surrounding tissues in the body [1–2]. It is known that there is an insufficient breakthrough in clinical treatment of cancer despite the progress in chemotherapy for years. There are still some limitations that restrain the efficacy and safety of cancer chemotherapy. The most important drawback is the poor aqueous solubility due to the hydrophobicity of most anticancer drugs [3]. Most of the anticancer drugs are formulated with co-solubilizers via intravenous administration; however these co-solubilizers lead to severe side effects restricting both the patient’s quality of life and efficacy of the therapy. Another major factor is that anticancer drugs have a wide distribution capacity in the body and owing to non-selective cytotoxicity of these drugs not only the cancer cells but also healthy cells are killed. Due to low therapeutic indices of anticancer drugs, a rapid increase and subsequent decay of drug concentration in blood is also one of the limitations of cancer therapy. Thus, the increase of drug concentration in blood during the chemotherapy falls into a decline in the cooling period which makes therapeutic effect repetition limited and therefore contributes to cancer cell growth.

Although in cancer therapy, the intravenous route is more common than the others, it is thought that with recent advances oral chemotherapy will be the breakthrough step in future chemotherapy [4]. From the patient’s viewpoint, the oral route allows for painless self-medication and thus it is considered the most convenient route. Besides, it reduces reimbursement load in the health budget also, since it does not require therapy in medical centres [5]. By means of oral administration, it is possible to prevent the initial rapid increase and the subsequent decay of drug concentration in blood that occurs via the intravenous route [6–7]. However most anticancer drugs are not good candidates for oral delivery owing to their low absorption in the gastrointestinal tract (GI) and as a result exhibit low oral bioavailability [8–9]. In order to develop an effective oral chemotherapy, the bioavailability of anticancer drugs should be improved [10].

Nanoparticulate drug delivery systems are promising in this field [11–12]. Nanoparticles are defined as submicron colloidal systems that include both nanospheres and nanocapsules. Nanospheres are defined as matrix systems whereas nanocapsules are core-shell structures consisting of an inner liquid core (which can be either aqueous or oily depending on the core material) surrounded by a polymeric wall [13–14]. Nanocapsules present many advantages, such as improving poor water solubility, maintaining drug stability by protecting the molecule from the environment, providing controlled release and improving the desired pharmacokinetic profile [15]. Considering the advantages of the nanocapsules depending on the structure, the oily core enables an improvement in the solubility of hydrophobic drugs and the polymeric wall surrounding it provides protection of the molecules against the harsh environment [16].

In cancer therapy, a common problem is drug ineffectiveness because of physicochemical and biopharmaceutical problems of drugs and CPT is cited as a prime example. CPT is a very effective anticancer agent against a wide spectrum of cancers such as colon, breast, ovarian, lung cancers in in vitro cell culture studies [17–18]. However its poor aqueous solubility and the pH-dependent stability problem results in the diminishing of clinical efficacy for the drug [19]. CPT is in active lactone form under pH 5 but is rapidly hydrolysed into the inactive carboxylate form in physiological or alkaline pH causing the effectiveness of chemotherapy to be reduced or even inhibited [20]. In addition to its stability problem, CPT has a very poor water solubility which is less than 1 µg/mL and is only soluble in dimethyl sulfoxide (DMSO) and mixtures of methanol with either dichloromethane or chloroform at a ratio 1:1 and 1:4 in volumes, respectively [21–22]. Therefore formulation approaches should be evaluated to improve the aqueous solubility and to protect this drug from hydrolysis at pH 7.4 in order to obtain an effective in vivo administration of CPT for cancer therapy. CDs can be a means of overcoming these problems for the in vivo behaviour of CPT upon intravenous or oral administration.

CDs are natural polymers which are produced from enzymatic degradation of starch [23]. They are cyclic oligosaccharides and consist of at least 6 D-(+)-glucopyranose units linked by α-(1,4) glucosidic bonds. The most common advantages of CDs in the pharmaceutical field are to enhance the stability, solubility, and bioavailability of drug molecules [24]. Amphiphilic CDs are derivatives of natural CDs which are chemically obtained and modified on the primary and/or secondary face [25]. It is very common to use CDs as complexing agents with the intention of increasing poor water solubility, maintaining stability, improving permeability and ultimately bioavailability [26]. Furthermore inclusion complexation with CDs can reduce or prevent GI irritation. It is known that complexation of drugs with CDs provide an improvement in its dissolution rate and consequently in oral absorption [27]. Besides, CDs serve as a permeability enhancer upon oral administration which plays an important role for drugs having low intestinal permeability [28–29].

Taking into consideration of the disadvantages that limit the effectiveness of oral chemotherapy and the advantages of nanoparticular drug delivery systems, developing a strategy with nanoparticles and even nanocapsules might be very promising for oral delivery of anticancer agents. Nanocapsules are especially beneficial for oral administration of anticancer drugs, e.g., CPT, suffering from poor solubility, instability, low permeability and ultimately poor oral bioavailability. Nanocapsules can i) enhance the poor solubility of hydrophobic drugs owing to the oily liquid cores, ii) improve the stability of drugs and prolong residence time in the GI tract due to the polymeric wall, and iii) enhance permeability of drugs by taking the advantages of both small size of nanoparticles and mucoadhesive properties of polymers as coating materials. The main goal of this study therefore in a first step, to design and evaluate nanocapsules for the oral delivery of the anticancer agent CPT, which has a limited clinical usage by maintaining its stability and improving its eventual bioavailability. For this reason, amphiphilic CD, 6OCAPRO (heptakis(6-*O*-hexanoyl)cyclomaltoheptose) was used as core polymer for nanocapsules. These nanocapsules were then coated with the cationic polymer chitosan to improve the cellular uptake and interaction with biological membranes and penetration through intestinal mucosa for absorption of CPT and reaching the circulation. Nanocapsules were characterized for their in vitro properties followed by cell culture studies evaluating anticancer efficacy and permeability in comparison to CPT in solution form.

## Results and Discussion

Nanoparticles take the advantages of small size in terms of both pharmacodynamic and pharmacokinetic profiles, e.g., release profiles, biodistribution, absorption rate and cellular uptake. Therefore the particle size should be in an optimum range which enables particles to diffuse and permeate through the biological membranes, but also should provide the maximum ability for encapsulation of drugs and sustained release. The influence of CD concentration, concentration of oil phase and organic to aqueous phase volume ratio have been studied to optimize the nanocapsule formulation (see Supporting Information File 1 for full experimental data).

The effect of the concentration of 6OCAPRO on particle size and PDI is given in Table 1. It is clearly seen that the particle size increases linearly with polymer concentration which is attributed to higher organic solution viscosity with increase in polymer concentration [30].

**Table 1:** Effect of polymer concentration on particle size and polydispersity index (PDI). Data represent the mean results ± SD values of three different batches.

Polymer concentration (% w/v)	Particle size (nm)	PDI ± SD

0.05	154.2 ± 3.2	0.10 ± 0.04
0.1	161.5 ± 3.7	0.11 ± 0.09
0.2	268.4 ± 4.0	0.19 ± 0.10
0.5	347.6 ± 5.6	0.32 ± 0.19

Although the smallest particle size was obtained with 0.05% w/v concentration of polymer, there is no significant difference between the concentrations of 0.05% w/v and 0.1% w/v (*P* > 0.05). Even if the polymer concentration increases two fold (from 0.05% w/v to 0.1% w/v,) the mean particle size does not increase two fold either. Herein providing stability might be an important factor at selecting the polymer concentration since polymeric shell has a key position for protection of encapsulated molecules and maintaining the stability. CD nanocapsules were prepared without a stabilizing agent, e.g., surfactant, which means drug stability is mostly yielded by the polymeric wall. Therefore the polymer concentration that is probably related with the polymeric wall thickness, is an important parameter in regard to give an idea for the thickness of this nanocapsule shell and for the stability thereby. Hence for further studies 0.1% w/v concentration of polymer was retained. PDI values indicated monodisperse distribution of particles and a higher polydispersity at increasing CD concentration.

This pre-formulation study was performed to select the optimum oil concentration with the goal of not only minimizing the particle size but also maximizing the capacity to dissolve the drug which is directly affected by the oily core of the nanocapsules. As shown in Table 2, an increase in the concentration of oil resulted in an increase of nanoparticle size. This effect was attributed to the increase of the viscosity of the organic phase, since the higher the oil concentration is, the more viscous the organic phase becomes. However there is no significant difference between the concentrations 0.3% v/v and 1% v/v (*P* > 0.05) in terms of mean particle size [31]. According to preliminary results a 1% v/v concentration of oil was selected for further studies.

**Table 2:** Effect of oil concentration on particle size and polydispersity index (PDI). Data represent the mean results ± SD values of three different batches.

Oil concentration (% v/v)	Particle size (nm)	PDI ± SD

0.3	179.2 ± 2.4	0.07 ± 0.02
1.0	182.5 ± 1.7	0.10 ± 0.04
3.0	296.0 ± 3.5	0.24 ± 0.10

The effect of increased aqueous phase volume was determined by investigating the particle size of nanocapsules using organic to aqueous phase volume ratio 1:1, 1:2 and 1:4. Table 3 shows that the mean particle size is unchanged with the organic to aqueous phase volume ratio 1:2 and 1:4 which means in terms of particle size there is no significance difference between these ratios (*P* > 0.05). On the other hand, the highest value of particle size was obtained with a lower volume of water (5 mL) which means, at the organic to aqueous phase ratio 1:1, a large increase in particle size was observed presumably due to the poor phase separation [32]. However as it was shown in a work performed by Simões et al., that decreasing the O/A ratio, in other words increasing the volume of the aqueous phase, results in a decrease of the percentage of the associated drug [33]. Therefore a 1:2 organic to aqueous phase volume ratio was selected for further studies.

**Table 3:** Effect of organic to aqueous phase ratio (O/A) on the particle size and PDI. Data represent the mean results ± SD values of three different batches.

O/A	Particle size (nm)	PDI ± SD

1:1	341.2 ± 4.7	0.31 ± 0.14
1:2	182.5 ± 1.6	0.09 ± 0.01
1:4	178.3 ± 2.5	0.07 ± 0.02

**Figure 1:**
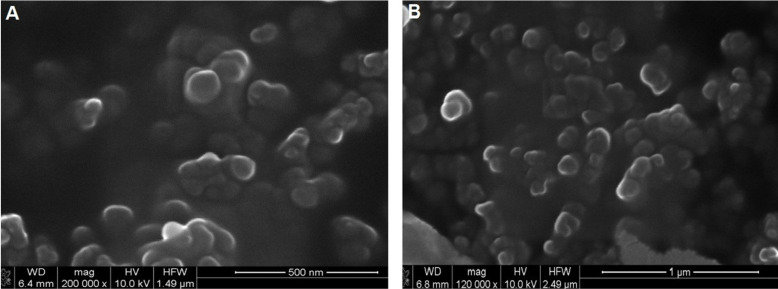
Scanning electron microphotographs of A) 6OCAPRO nanocapsules and B) CS-6OCAPRO nanocapsules.

According to the overall formulation development data, 0.1% w/v polymer, 1% v/v oil and 1:2 organic to aqueous phase volume ratios were selected as parameters for the CPT-loaded nanocapsule preparation. CPT-loaded CD nanocapsules were characterized in terms of mean particle size and size distribution, surface charge and morphology (see Supporting Information File 1 for full experimental data). As it can be clearly seen from the results (Table 4) both anionic and cationic CPT loaded nanocapsules were in the range of 150 to 250 nm with a narrow size distribution. Figure 1 shows SEM photographs of NCs with spherical shapes having smooth surfaces. SEM imaging is also a tool for justifying the results obtained from Zetasizer based on DLS measurements and it can be clearly seen that SEM photographs confirm the particle size are in nanometer range.

**Table 4:** Mean diameter, polydispersity index (PDI) and zeta potential values of CPT loaded nanocapsules. Data represent the mean results ± SD values of three different batches.

Formulations	Diameter (nm)	PDI	Zeta potential (mV)

6OCAPRO	187.50 ± 5.20	0.11 ± 0.04	−11.4 ± 1.2^a^
CS-6OCAPRO	204.20 ± 6.10	0.14 ± 0.06	+10.3 ± 0.7^a^

^a^Indicates a significant difference between formulations (*P* < 0.05).

The results of zeta potential analysis, also shown in Table 4, confirm that the anionic CD nanocapsules have a negative surface charge of −11 mV on the surface as compared with chitosan-coated cationic nanocapsules (CS-CD) that have a charge of +10 mV. The zeta potential of chitosan coated CD nanocapsules was significantly higher than uncoated CD nanocapsules (*P* < 0.05) due to the positive charge of CS based on its amino groups. Coating with CS resulted in an increment in particle size due to the adsorption of CS molecules on the nanocapsule surface [34]. This coating is believed to be of importance due to the penetration enhancing properties of chitosan through the intestinal mucosa by opening of tight junctions, as well as an expected synergistic anticancer effect coming from chitosan, a known caspase-3 activator [35–36].

The ability of nanocapsule formulations of different surface charge to entrap a drug was evaluated using CPT as a model drug (see Supporting Information File 1 for full experimental data). Table 5 represents the associated drug (%) and entrapped drug quantity (µg/mL) [37] in different nanocapsule formulations which were prepared in this study. As it can be seen from the results, the associated drug (%) of CD nanocapsules and CS-CD nanocapsules were found to be 46.9% and 50.7%, respectively. Chitosan-coated cationic CS-6OCAPRO nanocapsules have slightly higher entrapment efficiency compared with the anionic 6OCAPRO nanocapsules as also observed in Table 5.

**Table 5:** Associated drug (%), entrapment efficiency (%) and entrapped drug quantity (µg/mL) of 6OCAPRO and CS-6OCAPRO nanocapsules. Data represent the mean results ± SD values of three different batches.

Formulations	Associated drug %	Entrapped drug quantity (µg/mL)

6OCAPRO	46.96 ± 2.7	33.4 ± 1.1^a^
CS-6OCAPRO	50.70 ± 3.1	40.3 ± 0.7^a^

^a^Indicates a significant difference between formulations (*P* < 0.05).

It can be expected that coating the CD nanocapsule with CS will not result in a significant increase in drug loading since the lipophilic drug CPT will be largely encapsulated in the core of the nanocapsules with a smaller quantity adsorbed or entrapped in aliphatic chains of 6OCAPRO chitosan since this hydrophilic polymer is not an attraction site for the highly lipophilic drug molecule.

**Figure 2:**
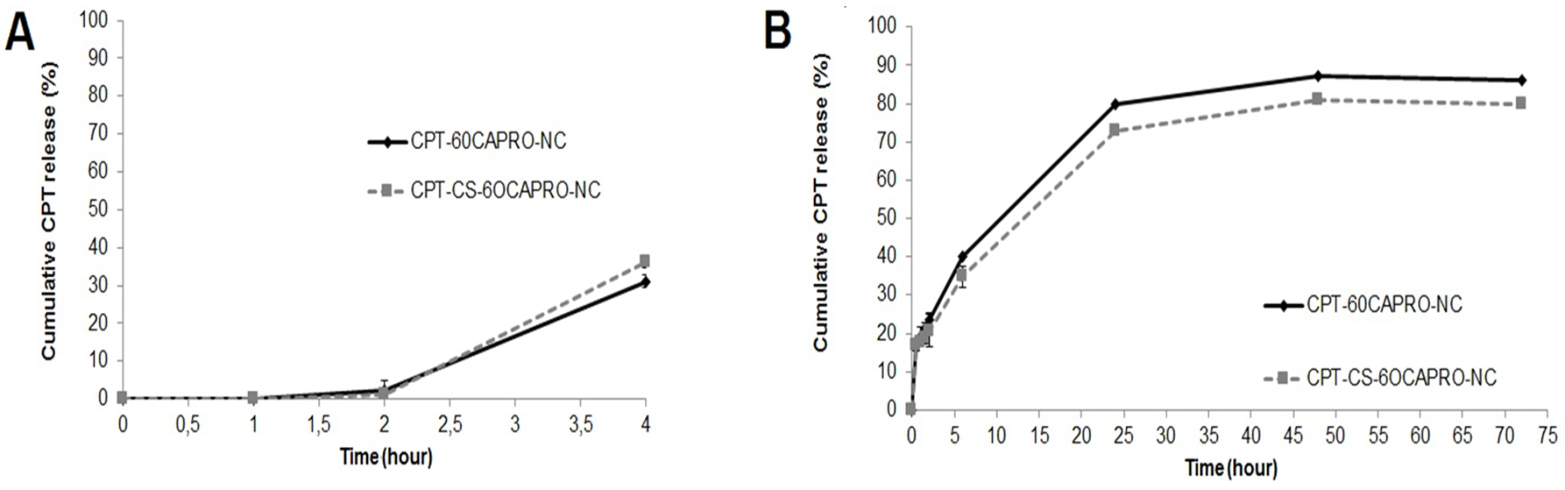
In vitro release profiles of CPT from anionic and cationic CD nanocapsules in pH 1.2 PBS A) in 2 hours and B) in pH 7.4 PBS in 72 hours. Data represent the mean results ± SD values of three different batches.

Oral administration of anticancer drugs is attractive in terms of reduced dosing frequency and preventing fluctuation in blood concentration. By means of nanocapsules, sustained release of the drug with prolonged systemic exposure can be achieved with minimum side effects by accumulation of the dosed drug in the tumour tissues at a desired rate by enhanced permeation retention effect (EPR) [3]. In vitro release of CPT from both anionic and cationic nanocapsules were performed in phosphate buffered saline (PBS) medium at pH 7.4 and at pH 1.2 to mimic the conditions inside the body representing blood and stomach, respectively (see Supporting Information File 1 for full experimental data). Figure 2 shows the cumulative percentages of CPT released from nanocapsules as a function of time. As it is shown in Figure 2A, we did not observe any CPT release from nanocapsules in the first 2 hours (which represents the residence time in stomach). This can be attributed to the polymeric wall of nanocapsules that protect particles from the acidic environment of the stomach. On the other hand for the release medium pH 7.4; the release profiles indicated that in the first 2 hours approximately 25% of CPT was released from the formulations and complete release of encapsulated drug was found within a period of 72 hours. Results indicate that both of the formulations exhibited a sustained release profile. This observation can be explained by the slow release of CPT from the nanocapsules. These results suggest favourable release behaviour for an effective cancer treatment since nanocapsules would accumulate within the tumour tissue and release the drug in a sustained manner over time [38]. In addition, anionic CD nanocapsules released CPT slightly faster than chitosan-coated cationic CD nanocapsules. Faster release of CPT from anionic nanocapsules can be attributed to the smaller particle diameter and higher drug loading in these formulations. It was also previously reported that drug release rates from large nanoparticles were slower than the release profile from smaller nanoparticles [16,39]. This is quite understandable since smaller particle sizes have a larger surface area per unit mass or volume, and the increased surface area provides more opportunities in drug delivery. Thus, CPT that is adsorbed on the surface of nanoparticles shows a release profile with an initial burst effect when suspended followed by the diffusion of CPT from the oil core to the release medium [40].

Oral drug delivery systems should be designed to protect their therapeutic load from the harsh environment and wide pH range through the GI tract. Therefore since the stability of CPT under GI conditions was already demonstrated in Figure 2A,B, the stability of the nanocapsules was also evaluated in SGF and SIF to determine whether nanocapsules maintain their physicochemical properties like size or zeta potential in the gastric and intestinal environment.

In order to check the GI stability of nanocapsules, formulations were incubated with simulated GI media and the changes in mean particle size, polydispersity index and zeta potential values before and after incubation were measured (see Supporting Information File 1 for full experimental data). The effects of simulated gastric fluid SGF and simulated intestinal fluids on the CPT stability are shown in Table 6. According to the results it can be seen that both anionic and cationic nanocapsules were found to be stable in simulated gastric and simulated intestinal fluids. This formed an important finding as the drug and the nanocapsules must remain stable in GI fluids until they reach absorption areas [41]. In this work stability of CPT was provided by the protective effect of nanocapsules and physically stable nanoparticles would increase the consequential efficacy of CPT due to increased residence time in GI tract as well as better uptake and absorption of the drug [4].

**Table 6:** Stability of various CPT formulations in simulated GI fluids.

Formulations	Parameters	Mean particle size (nm)		PDI		Zeta potential (mV)	
		Initial	Final	Initial	Final	Initial	Final

6OCAPRO	SGF^a^ pH 1.2	187.3	189.4	0.091	0.121	−10.4	−5.92
	SIF^b^ pH 6.8	187.3	199.7	0.091	0.175	−10.4	−7.68
CS-6OCAPRO	SGF pH 1.2	197.8	185.9	0.102	0.123	+17.1	19.20
	SIF pH 6.8	197.8	203.7	0.102	0.115	+17.1	18.45

^a^Simulated gastric fluid and ^b^simulated intestinal fluid.

**Figure 3:**
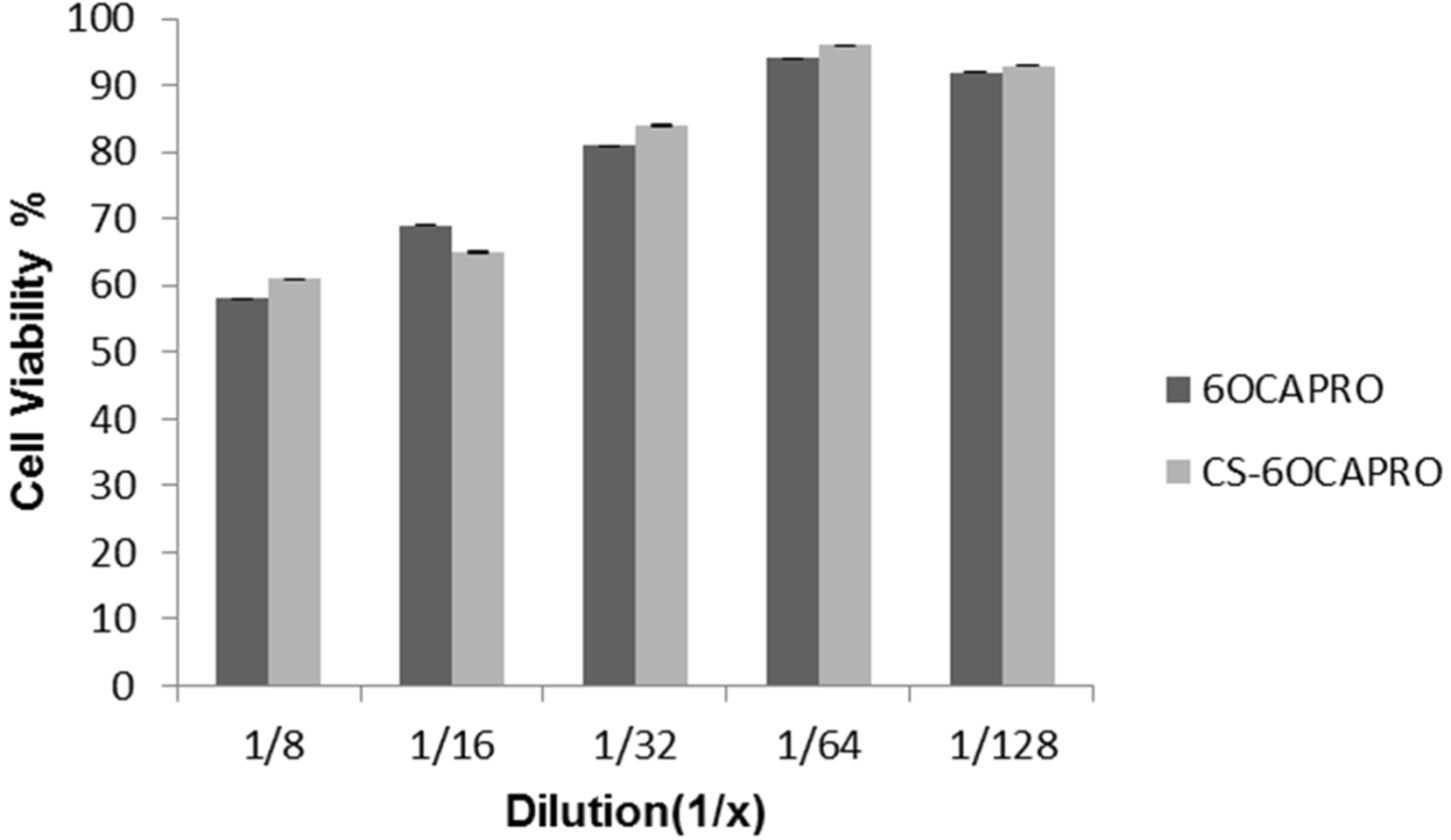
Cell viability of blank 6OCAPRO and CS-6OCAPRO nanocapsules against L929 cells after 48 h incubation. Data represent the mean results ± SD (*n* = 4).

The L929 cell line is the recommended cell line by the USP to test toxicity of polymeric systems and thus used in this study to investigate whether the toxicity of nanocapsules is associated with the polymer material itself or not. Therefore cytotoxicity of blank anionic and cationic CD nanocapsules was evaluated against L929 cells with an MTT assay (see Supporting Information File 1 for full experimental data). Figure 3 shows the cell viability of L929 mouse fibroblast cells after 48 hours of incubation with unloaded CD nanocapsules at dilution rates of 1:8, 1:16, 1:32, 1:64 and 1:128. No significant difference was observed between anionic and cationic CD nanocapsules in terms of cell viability values for all the concentrations tested (*P* > 0.05). It is also seen that the toxicity of blank nanocapsules were concentration dependent and from the dilution rate 1:16 v/v (concentration is 3.125 µg/mL) on, blank nanocapsules were found to be safe.

**Figure 4:**
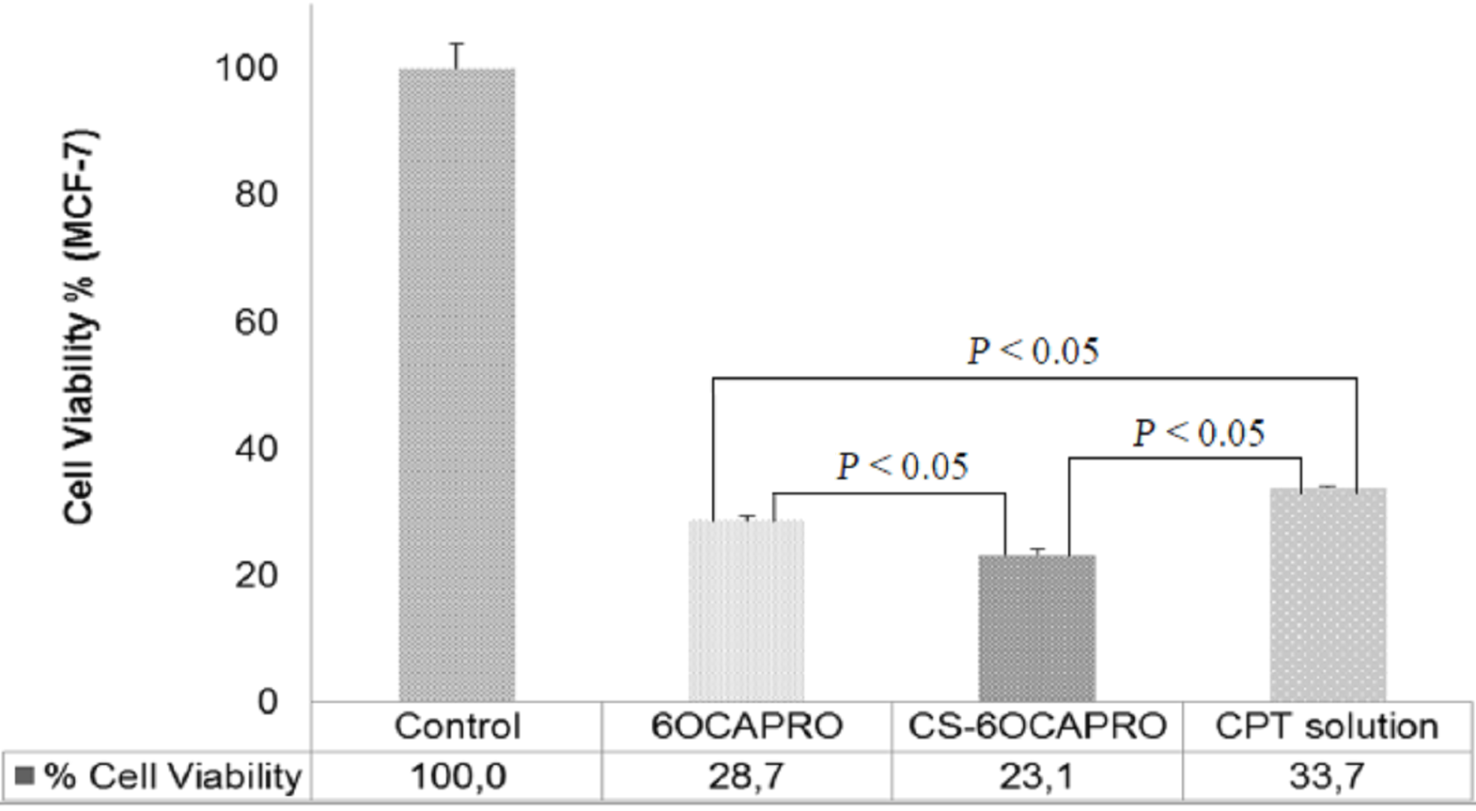
Viability of MCF-7 cells cultured with CPT loaded 6OCAPRO and CS-6OCAPRO nanocapsules in comparison with CPT in solution form at the same concentration. Data represents the mean results ± SD (*n* = 4). *P* < 0.05 indicates a significant difference between formulations.

Figure 4 shows the viability of MCF-7 cancer cells after 72 hours of incubation with the formulations diluted with DMSO at a dilution rate of 1:16. The cytotoxic effect of both anionic and cationic nanocapsule formulations was investigated in comparison with CPT solution (CPT in DMSO) at a concentration of 3.125 µg/mL (see Supporting Information File 1 for full experimental data). The incubation time is depending on both the doubling time of the MCF-7 cell line and the release characteristics of the formulations. The MCF-7 cell line has a doubling time over 38 h and CPT was released from nanocapsules within a period of 72 hours in a sustained manner which means the accumulation into the cells needs a prolonged exposure. Moreover, CPT, as a topoisomerase I inhibitor, has the inhibitory activity in the S-phase of the cell cycle [42]. Therefore prolonged exposure of CPT is necessary to increase cell death while the S-phase is a short phase of the cell cycle and both pre-clinical and clinical studies revealed that prolonged exposure of CPT and analogs brings about a more effective therapy [43].

Results indicate that both of the CD nanocarrier systems caused more cancer cell mortality than CPT in DMSO which means there is a significant difference between the formulations and free CPT (*P* < 0.05). Furthermore chitosan-coated cationic nanocapsules showed a significantly higher cytotoxic effect compared with the anionic nanocapsules (*P* < 0.05). This increasing cytotoxic effect can be attributed to the surface charge of the coating material chitosan. CS coated cationic NCs exhibited a stronger affinity for the negatively charged cell membrane [44]. CS can increase the chance of cellular uptake of nanoparticles by improving the residence time at the cellular surface due to the electrostatic interaction between the cell membrane and nanoparticle since positively charged CS has the ability to interact with the negatively charged surface membrane of cells via electrostatic forces [45].

**Figure 5:**
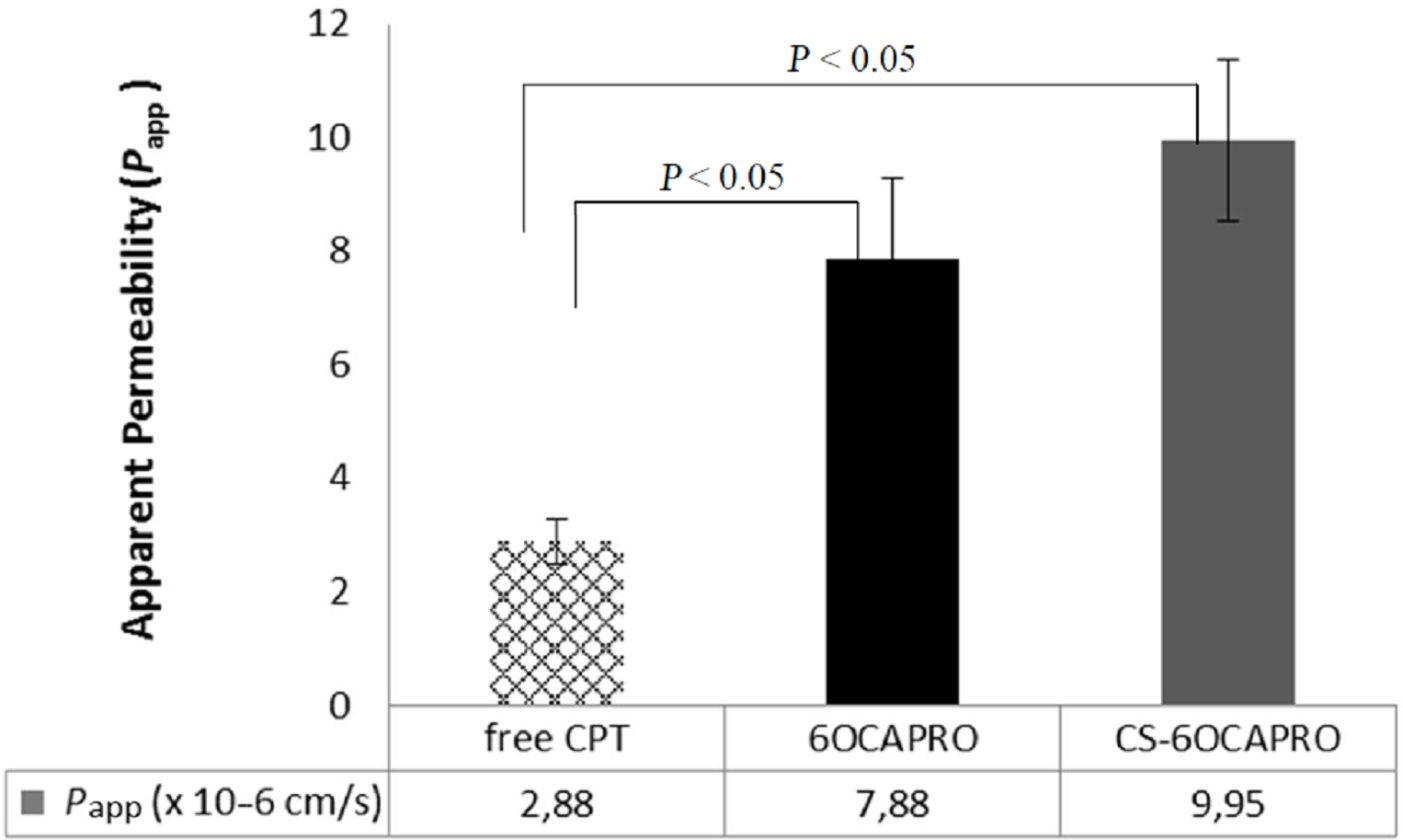
Apparent permeability coefficient (*P*_aap_) of different CPT formulations: CPT in DMSO solution, CPT loaded 6OCAPRO and CS-6OCAPRO nanocapsules. Data represent the mean results ± SD (*n* = 4). *P* < 0.05 indicates a significant difference between formulations.

The permeation of CPT both in solution form and in nanocapsule formulations across a Caco-2 cell monolayer is demonstrated by the apparent permeability coefficients in Figure 5 (see Supporting Information File 1 for full experimental data). Results indicate that in terms of permeability coefficients, there is a significant difference between the formulations and free CPT (CPT in DMSO) (*P* < 0.05). Encapsulation of CPT in nanocapsules 3 fold increased the CPT transport compared with free CPT. Despite the fact that the particle size plays a critical role for the penetration through membranes, free CPT molecules showed a significantly lower permeability compared with CPT-loaded CD nanocapsules which is probably attributed to the lactone–carboxylate equilibrium of CPT. It is known that the active lactone form of CPT rapidly hydrolyses into the inactive carboxylate form at physiological pH. As it was indicated in Supporting Information File 1, all the formulations were diluted with HBSS solution at pH 7.4 for the permeability studies. At this pH, the active lactone form which is essential for the diffusion of the drug through membranes, was rapidly turned into the inactive carboxylate form which shows a poorer diffusibility than the lactone form [46–47]. Nanocapsules have the ability to maintain the drug stability of CPT at this pH by reducing the rapid hydrolysis and probability of the occurrence of the carboxylate form. Hence encapsulation of CPT in nanocapsules results in an enhancement of the lactone form rate thereby improving the permeability. Moreover, CDs as absorption enhancers play also an important role in the improvement of CPT permeability when formulated with nanocapsules.

Although the differences between anionic and cationic formulations are not significative (*P* > 0.05), chitosan-coated cationic nanoparticles were able to penetrate more into cells than anionic ones probably due to higher cellular adhesion and increasing residence time at the cell surface provided by CS molecules. This finding suggests that with chitosan-coated cationic NCs, electrostatic interactions between positively charged CS amino groups and the negatively charged cell membrane occurred. Therefore, the penetration of CS-coated cationic CD NCs was greater than the anionic CD NCs [48–50]. Hence an improved permeability might be a first step in predicting the blood concentration of the drug with appropriate pharmaceutical effect [51].

## Conclusion

In this work CPT-loaded amphiphilic CD nanocapsules were developed and in vitro evaluated for oral chemotherapy for the first time. The results obtained from this work suggest that CD nanocapsules offer a new strategy for the development of a safe and effective oral chemotherapy. CD nanocapsules might be an interesting strategy to improve the stability and bioavailability of the anticancer drug CPT. In a second step, interaction with mucus and the GI uptake of nanocapsules will be evaluated using artificial mucus model and in vivo animal studies.

## Supporting Information

The Supporting Information includes pre-formulation studies of CD nanocapsules, physicochemical characterization of CPT-loaded CD nanocapsules, determination of CPT loading, in vitro CPT release studies, CPT stability in simulated GI fluids and cytotoxicity, anticancer efficacy and permeability assay of CD nanocapsules.

File 1 Experimental data.
