# Supplementary material for: Formulation development, stability and anticancer efficacy of core-shell cyclodextrin nanocapsules for oral chemotherapy with camptothecin
Source: Beilstein J Org Chem. 2015 Feb 4;11:204–12. doi: 10.3762/bjoc.11.22 (PMC4362320; doi:10.3762/bjoc.11.22)
Supplement: File 1 — Experimental data. [file Beilstein_J_Org_Chem-11-204-s001.pdf]

Supporting Information

for

Formulation development, stability and anticancer efficacy of core-shell cyclodextrin nanocapsules for oral chemotherapy with camptothecin

Hale Ünal[1], Naile Öztürk[2] and Erem Bilensoy*[1,2]

Address: [1]Division of Nanotechnology and Nanomedicine, Graduate School of Science and Engineering, Hacettepe University, Beytepe, Ankara, 06800, Turkey and [2]Department of Pharmaceutical Technology, Faculty of Pharmacy, Hacettepe University, Sıhhıye, Ankara, 06100, Turkey

Email: Erem Bilensoy - eremino@hacettepe.edu.tr

*Corresponding author

Experimental data

Pre-formulation studies of CD nanocapsules

Core-shell nanocapsules were prepared by a modified nanoprecipitation method. Briefly, an organic solution of amphiphilic CD (MW: 1820 g/mole, Sevilla University) (concentration range 0.05% w/v to 0.5%, w/v) and oil Miglyol 812 (Condea Chemie, Germany) (concentration range 0.3% v/v to 3% v/v) in ethanol (5 mL) was added drop wise into ultra-pure water (5, 10 or 20 mL) under moderate magnetic stirring.

Organic solvent was then evaporated under vacuum at 40 °C for 30 min to obtain nanocapsule dispersion at the final desired volume 10 mL. Nanocapsules were recovered by centrifugation at 3500 rpm at room temperature for 15 min. Pre-formulation studies were performed to determine the effect of formulation parameters such as oil concentration, ratio of organic to aqueous phase and CD concentration on nanoparticle characteristics such as mean diameter, polydispersity and zeta potential. According to the pre-formulation results, optimum formulation parameters were selected and CPT (95% HPLC powder, MW: 348.35 g/mole, Sigma-Aldrich) loaded nanocapsules were prepared with the same parameters dissolving CPT (10% w/w of the polymer) in organic phase. To obtain positively charged nanocapsules 0.025% w/v of Protasan™ (MW < 200 kDa, Novamatrix) was added to aqueous phase.

Physicochemical characterization of CPT loaded NCs

Particle size and zeta potential

Mean particle size and polydispersity index (PDI) of the CPT-loaded CD nanocapsules were determined by dynamic light scattering (DLS) (Malvern Zetasizer Nano ZS series, UK) at a scattering angle of 173°. Zeta potential of nanocapsules was measured in mV with Malvern Zetasizer Nano ZS at an angle of 12° and 25 °C. Each measurement was carried out in triplicate.

Imaging of nanocapsules

The morphology of nanocapsules was determined by using Scanning Electron Microscope (FEI Nova™ Nano SEM 430, ABD). Samples were mounted on the metal

stubs and coated with 100 Å thick layer of a Gold-Palladium alloy. Then the particles were imaged at 5 to 20 kV.

Determination of CPT content in nanocapsules

Content of CPT in the nanocapsules was quantified by a previously validated HPLC method equipped with a fluorescence detector at an emission wavelength (λ_{em}) of 435 nm and at an excitation wavelength (λ_{ex}) of 370 nm. (Agilent 1100, Germany). Briefly, after the separation of unbound drug by centrifugation at 3500 rpm for 15 min, supernatant was separated and freeze-dried in order to obtain drug loaded nanoparticle in powder form. Resulting powder containing CPT loaded nanocapsules (1 mg) was dissolved in 2 mL of a mixture of DCM and DMSO. The experimental CPT loading was quantified using the peak area of each NC formulation.

Associated Drug (%) and Entrapped Drug Quantity (µg/mL) were calculated as follows; [1].

$$\text{Associated Drug (\%)} = \frac{\text{Experimental Drug Loading}}{\text{Theoretical Drug Loading}} \times 100 \qquad (1)$$

$$\text{Entrapment Drug Quantity} = \frac{\text{CPT quantity (µg)}}{\text{Formulation volume (mL)}} \qquad (2)$$

In vitro release studies of CPT from nanocapsules

Release behaviour of CPT from nanocapsules was carried out at 37 °C with dialysis membrane (Cellulose Membrane MW: 100,000 Da, Sigma Chemicals., USA) technique under sink conditions. 2 mL of nanocapsule dispersion was put up in a

dialysis bag. The bag containing the nanocapsule suspension was placed in 50 mL of phosphate buffer solution (PBS) containing 0.1% (v/v) Tween 80 at pH 7.4 and pH 1.2 to mimic the physiological conditions. The system was placed in a shaking water bath at 37 °C with an agitation speed of 100 rpm. At specific time periods (0,5., 1., 1,5., 2., 6., 24., 48. and 72.hours), 500 µL of sample was taken from the medium and replaced by the same volume of fresh medium. The cumulative percentage of drug released for each time point was calculated as a percentage of the total drug incorporated into the nanocapsules.

Stability of NCs in simulated gastrointestinal fluids (SGF &SIF)

It is also necessary to determine the physical stability of drug loaded nanocapsules under the conditions of the GI tract. Nanocapsules are bound to encounter during GI transit with a wide pH range to better elucidate the in vivo behaviour and anticancer efficacy of the nanocapsules. With oral administration, nanoparticles should maintain the stability of the encapsulated drug during the residence time in GI tract before reaching the absorption region in the intestines. The stability of the nanoparticles and encapsulated drug herewith, are mostly effected by the pH dependent environment of gastrointestinal regions. To test the protective effect of this polymeric shell of nanocapsules on the stability of the encapsulated drug CPT, simulated gastric and simulated intestinal fluids were used as media. All the formulations were incubated in both simulated gastric fluid (SGF) pH 1.2 for 2 hours and simulated intestinal fluid (SIF) pH 6.8 for 6 hours. These pH dependent stability studies were carried out in the following media by measuring the mean particle size, polydispersity index and zeta potential values of nanoparticles. SIF was prepared by dissolving 680.5 mg monobasic potassium phosphate (KH_2PO_4) in 100 mL deionized water. 89.6 mg of

NaOH was added to adjust the pH of the media. SGF without enzyme was prepared by dissolving 100 mg of NaCl in 50 mL deionized water containing 0.35 mL concentrated HCl. For each formulation; 1 mL of nanoparticle dispersion was added to 9 mL of simulated media and incubated in for 2 hours for SGF and for 6 hours for SIF. Mean particle size, PDI and zeta potential values were measured before and after incubation.

Cytotoxicity of blank CD NCs against L929 cell line

The cytotoxicity of blank CD nanocapsules was determined on mouse fibroblast cells with MTT assay. L929 (American Type Culture Collection, USA) was cultured in Dulbecco's modified Eagle's medium (DMEM) containing 10% fetal bovine serum (FBS), penicillin (50 units/mL) and streptomycin (50 µg/mL) in 25 cm^2 culture flasks. L929 fibroblast cells were seeded at a density of $5x10^3$ cells/well in 100µl DMEM. The cultures were incubated at 37 °C in an incubator humidified with 5% CO_2. (Humidified CO_2 is used to prevent the cell from drying by mimicking the physiological conditions.) After 24 hours cell culture medium was replaced with fresh medium containing blank anionic and blank cationic CD nanocapsules with different dilution rates (1:8, 1:16, 1:32, 1:64 and 1:128). After 48 h of incubation, 25 µL of MTT solution in PBS (5 mg/mL) were added to each well and were incubated for further 4 h. MTT was aspirated off and then 200 µL of DMSO was added onto it. Optical densities (OD) were determined by a microplate reader (Molecular Devices, USA) at 560 nm. Cells incubated in culture medium alone served as a control for cell viability (nontreated wells). Cell viability (%) was calculated as (OD of treated wells/OD of nontreated cells) × 100.

Anticancer efficacy of CPT loaded CD NCs against MCF-7 cell line

The anticancer efficacy of CPT loaded nanocapsules was determined against MCF-7 cell line in comparison to CPT solution with MTT assay. MCF-7, human breast adenocarcinoma cell lines were obtained from the American Type Culture Collection (ATCC, LGC Promochem, Rockville, MD, USA). Cells were cultured in Dulbecco's Modified Eagle Medium (DMEM) supplemented with 10% fetal bovine serum (FBS), penicillin (50 units/mL) and streptomycin (50 µg/mL). The cultures were maintained at 37 °C in a humidified 5% CO_2 incubator. MCF-7 cells were seeded in 96- well plates at a density of 1×10^4/100 µL per well and incubated for 24 hours to allow the cell attachment. The cells were incubated with CPT loaded 6OCAPRO and chitosan coated CPT loaded 6OCAPRO nanocapsules and CPT in DMSO solution for 72 h. All the formulations were diluted with DMEM at a dilution rate 1:16 v/v (an equivalent dose of CPT at 3.125 µg/mL) At determined time, formulations were replaced with 25 µL of MTT solution and cells were then incubated for an additional 4 h. MTT was aspirated off and in order to dissolve formazan crystals, 200 µL DMSO were added onto it. Optical density (OD) was measured at 560 nm using a microplate reader (Molecular Devices, USA). Untreated cells (cells incubated with only culture medium) were taken as control with 100% cell viability. Experiments were performed in triplicate and results are expressed as mean ±SD.

In vitro transport studies across Caco-2 cell line

In vitro transport studies were carried out across the human adenocarcinoma Caco-2 cell line, which is a widely used model for intestinal drug absorption and mimic the gastrointestinal barrier for oral chemotherapy in the recent years.

Caco-2 cells with a passage number 30–35, were cultured on polycarbonate membrane filters (Thincerts ™ transparent, pore size 1 μm, area 1,13 cm^2) in 12-well plates (Greiner bio-one, Germany) at a seeding density of 6 × 10^4 cells in each well. The culture medium was added to both the apical (500 μL) and basolateral (1 m L) of filter insert and was replaced every other day during 20 days. The cells were left at 37 °C in an incubator under atmosphere of 95% air and 5% CO_2 at 90% relative humidity. Before the experiments, cell monolayers were replaced with transport buffer containing Hank's balanced salt solution (HBSS) buffered with 10 mM n-(2-hydroxyethyl) piperazine-n-(2-ethanesulfonic acid) (HEPES) at pH 7.4 and the trans epithelial electrical resistance (TEER) of monolayers was measured by using a Millicell®-ERS-2 system (Millipore Corporation, Bedford, MA) and found to be between 200-300 Ω cm^2. The transport of CD nanoparticle formulations (6OCAPRO and CS-6OCAPRO) and CPT solution in DMSO were studied from the apical to basolateral direction in Caco-2 cells. All the test solutions were diluted with HBSS in order to evaluate CPT concentration at 25 µg/mL both in nanocapsules and in solution form. Each experiment was started by adding 0.5 mL of test solution to the apical side and 1 mL of HBSS to the basolateral side of the Transwell inserts. The cells were then incubated at 37 °C and 30 rpm for 4 hours. Samples were taken from the basolateral side and analysed with a validated HPLC method. Apparent permeability coefficient (P_{app}) which is expressed in centimetres per second was calculated according to the formula (3)

$$P_{app} = \frac{dQ}{dt} \text{ x } \frac{1}{A\,Co} \qquad (3)$$

where dQ/dt is the permeability rate, Co is the initial concentration on the apical side and A is the surface area of the monolayer.

Statistical analysis

The results were expressed as mean ± SD. The significance of differences was estimated by Student's *t*-test. P value less than 0.05 ($P < 0.05$) was considered to indicate a statistically significant difference.

References

[1] Abbasi, S.; Paul, A.; Shao, W.; Prakash, S. *J. Drug Delivery.* **2012**, *2012*, 1-8
